# Neurophysiologic evidence for increased retrieval suppression among negative ruminators

**DOI:** 10.1002/brb3.1748

**Published:** 2020-08-03

**Authors:** Aarti Nair, Joshua C. Eyer, Mark E. Faust

**Affiliations:** ^1^ Department of Psychology School of Behavioral Health Loma Linda University Loma Linda CA USA; ^2^ Capstone College of Nursing University of Alabama Tuscaloosa AL USA; ^3^ Department of Psychological Science University of North Carolina Charlotte NC USA

**Keywords:** emotional valence, ERP, memory suppression, no‐think, rumination, think

## Abstract

**Introduction:**

Events (e.g., seeing a familiar face) may initiate retrieval of associated information (e.g., person's name), but not all cue‐initiated memory retrieval is welcome (e.g., trauma). Retrieval suppression refers to the ability to halt unwanted retrieval, and any erosion of memory associations in response to repeatedly excluding a memory from consciousness. The current study sought to examine event‐related potential (ERP, averaged scalp electrical recordings) correlates of inhibitory cognitive control of memory retrieval and any linkage of such control to ruminative memory styles.

**Methods:**

Participants (*N* = 23) first learned face‐picture pairings. ERPs were then recorded as they viewed face cues while either bringing the associated picture to mind (think trial), or not allowing the associated picture to come to mind (no‐think trial).

**Results:**

Emotional valence of learned pictures (negative versus neutral) modulated a posterior (P1, 100–150 ms) ERP associated with attention to the face cue. Memory strategy (think versus no‐think) modulated a frontal (P3, 350–450 ms) associated with alerting of the need to control retrieval. Both valence and strategy worked in combination to modulate a late posterior (LC, 450–550 ms) ERP associated with successful memory retrieval. Brooding, a negative form of rumination, was found to be positively correlated with the LC ERP.

**Conclusion:**

The results suggest early separation of emotional and strategic control of retrieval, but later combined control over access to working memory. Moreover, the positive correlation of brooding and the LC suggest that individuals who are high in application of perseverative strategies to memory retrieval will show greater modulation of the retrieval‐related LC ERP.

## Graphical abstract

Most individuals are able to keep unwanted memories out of everyday consciousness to get through their daily activities. This adaptable control process helps us manage our emotional memories. However, this process may be disrupted in individuals experiencing psychiatric conditions characterized by intrusive ruminative thoughts, such as post‐traumatic stress disorder. Here, we use electroencephalography techniques to demonstrate that our participants are able to successfully learn to suppress negative stimuli. Our findings suggest that there may be potential ways to enhance retrieval suppression mechanisms for individuals with psychiatric conditions before their symptoms become debilitating.

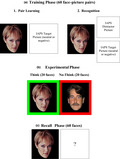

## 1 INTRODUCTION

The *executive deficit hypothesis* (Levy & Anderson, 2008) suggests that not only is our ability to correctly retrieve memory, but the ability to halt unwanted retrievals, perhaps automatically cued by stimulus events, is of critical importance to a proper functioning memory system. The main idea is that retrieval suppression of unwanted memories is a key cognitive ability.

Imagine that as you stand in line for a morning cup of coffee you realize the person in line in just front of you was someone you first met at a recent social gathering. Seeing this person cues retrieval of several memories from that gathering, but you are disappointed that memory of the person's name is not recalled. You are further dismayed to find that memory of an embarrassing moment at the gathering you would rather not think about has been cued and retrieved. While much research effort has gone into studying successful cued recall, there is an increasing recognition that it is not uncommon for cues to initiate retrieval of memories we would rather not think about (Anderson & Levy, 2009; Levy & Anderson, 2002). Moreover, trauma and mood disorders often result in increased ruminations involving negative memories (Nolen‐Hoeksema, 2000; Nolen‐Hoeksema & Morrow, 1991). The present study provides important support for the executive deficit hypothesis (Levy & Anderson, 2008) by assessing the relationship between event‐related potential (ERP) correlates of retrieval suppression and ruminative behaviors predicted by the executive deficit hypothesis (Levy & Anderson, 2008). We also seek to replicate and extend ERP correlates associated with retrieval suppression to support further development of our understanding of the neural basis of control over intrusive memories associated with clinical disorders such as PTSD and depression (Gagnepain, Hulbert, & Anderson, 2017).

### 1.1 Assessing retrieval an memory suppression effects

To study this problem, Anderson and Green (2001) developed a procedure (think/no‐think task) where participants first learned cue‐target pairings to a criterion level, followed by a second memory control phase where they were presented with a cue item and instructed to either allow the associated target item to come to mind (i.e., think trials) or to *not* allow the target item to come to mind (i.e., no‐think trials). Cue‐target pairs are typically repeated several times during this phase. Of interest is the extent to which effortful cognitive control can modify the operation of automatic cued retrieval (Anderson & Levy, 2009) in (a) a transitory manner by reducing conscious awareness of the no‐think cued items, and (b) in a more durable way by weakening links in memory to no‐think cued items. Both of which are often referred to as memory suppression, and the one is thought to lead to the other (Anderson & Levy, 2009).

In lieu of methods to directly measure the contents of consciousness, the standard think/no‐think task includes a final cued recall test. The largest most robust effect (*total control effect*) observed is that final cued recall is greater for think than for no‐think cue‐target pairs. A smaller, less robust effect (*negative control effect*) is assessed by holding back some of the initially learned cue‐target pairs from the memory control phase to act as baseline comparison pairs. Observation of reduced final cued recall for no‐think pairs, in relation to baseline pairs (negative control effect), has been argued to be an indication of suppression of links to cued target item representations (Anderson & Levy, 2009; Levy & Anderson, 2002). Negative control effects are not always observed in variations of the think/no‐think task (e.g., Bulevich, Roediger, Balota, & Butler, 2006), and several studies (e.g., Dieler, Herrmann, & Fallgatter, 2014) have explored factors that decrease the likelihood of observing a negative control effect.

The idea that repeated transitory overriding of automatic cued retrieval during no‐think trials of the think/no‐think task (*retrieval suppression*) can result in durable reductions in the ability of subsequent cued retrieval highlights the importance of cognitive control of memory (Anderson & Levy, 2009). Evidence has accumulated for a neural system for downregulation of intrusive memories via dorsolateral prefrontal control over the hippocampal complex (Anderson & Green, 2001; Anderson & Levy, 2009). Levy and Anderson (2008) proposed that individual differences in the effectiveness of neurocognitive control over automatic cued retrieval of unwanted memories of traumatic events may play an important part in recovery following trauma.

One method of assessing transitory effects of cognitive control during no‐think trials is to use EEG recordings to isolate event‐related potentials (ERPs) associated with conscious memory retrieval during performance of memory control (i.e., think versus no‐think) trials in the think/no‐think paradigm. Bergstrom and colleagues (Bergstrom, Fockert, & Richardson‐Klavehn, 2009; Bergstrom, Velmans, Fockert, & Richardson‐Klavehn, 2007) reported an early anterior ERP associated with memory control strategy (think versus no‐think), and a late posterior ERP associated with successful retrieval of the cued item. Their results suggest that the think versus no‐think differential in the late retrieval‐related ERP can be used to index transitory retrieval suppression.

### 1.2 Memory suppression and emotionally valenced items

Much of the early work using the think/no‐think task used randomly paired words to form cue‐target pairs learned during the initial phase of the task. To improve the external validity of the think/no‐think task in applied settings, a version using emotionally valenced objects and scenes as target items, and photographs of faces as memory cues was developed by Depue, Banich, and Curran (2006). Results yielded memory suppression effects (i.e., negative control effects, no‐think minus baseline final cued recall) that were greater for negatively valenced stimuli than neutrally valenced stimuli. Lambert, Good, and Kirk (2010) found a similar greater suppression effect for negatively valenced word pairs (e.g., cruel‐socks) versus positively valenced word pairs (e.g., joy‐socks).

### 1.3 Neural basis of retrieval suppression

While the neurobiological basis of durable memory suppression (e.g., negative control effects in the think/no‐think task) remains unknown, the neural networks and neural dynamics associated with retrieval suppression (e.g., no‐think trials in the think/no‐think task) have been explored.

#### 1.3.1 Neural Network Supporting Retrieval Suppression

Studies using fMRI to image brain regions involved during performance of the think/no‐think task (unrelated cue‐target neutrally valenced word pairs; Anderson & Green, 2001) suggest that dorsolateral prefrontal networks send control signals to regulate temporal lobe memory access and the activity of modality‐specific cortical representations. Depue, Curran, and Banich (2007; neutral face cues paired with negative scenes) reported evidence for an early inferior frontal gyrus initiated regulation of hippocampus (memory retrieval) and amygdala (emotional content processing), and a slower medial frontal gyrus regulation of visual cortical areas supporting visual awareness of the retrieved target picture. They also suggested that the frontal poles may be involved in regulation of posterior cortical representations of emotional content of retrieved memory representations. Gagnepain et al. (2017) used similar stimuli (face cues paired with target scenes of either neutral or negative emotional valence) and a newer enhanced version of the think/no‐think task combined with fMRI imaging and effective connectivity analysis that allows identification of neural regions have on each other in complex networks. Their results suggested that medial frontal gyrus (as part of a larger fronto‐parietal network) was the likely source of parallel downregulation signals to hippocampus (cued retrieval), amygdala (emotional aspects of cued scene), and parahippocampal cortex (visual features of cued scene).

#### 1.3.2 Neuroelectric correlates of retrieval suppression (neutral word pairs)

Documentation of a retrieval‐related ERP (posterior, often larger over the left hemisphere, 400 to 800 ms; Rugg, 1995) has been of central interest to EEG study of retrieval suppression. This effect has also been found to be absent in patients with impaired recollection due to hippocampal lesions (Duzel, Vargha‐Khadem, Heinze, & Mishkin, 2001), and it has been suggested to originate from recollection‐related activity in hippocampal‐parietal cortical networks (Curran, Tepe, & Piatt, 2006).

Bergstrom et al. (2007) provided the first ERP study of the think/no‐think task (unrelated neutrally valenced word pairs). Both think and no‐think ERP trials were segregated into recalled and unrecalled subcategories, based on the subsequent final cued recall test. This allowed identification of memory control ERPs (think versus no‐think difference) from memory retrieval ERPs, involving an interaction of memory control and learning status. The results yielded an early memory control ERP (i.e., think versus no‐think) in the 200–300 ms time window with spatially and temporally separable anterior and posterior components. The anterior component was proposed to be associated with selection of memory control strategy based on color (red versus green) of the cue word presented, and the posterior component was proposed to be involved in attentional selection of the critical visual feature of the cue word (red versus green) that would indicate the correct memory strategy for that trial.

Bergstrom et al. (2009) extended these findings by documenting a later (300–500ms) additional anterior ERP that was observed only for participants instructed to use a strict memory suppression strategy. They argued this ERP was associated with later conscious memory control strategies. In sum, the early studies support (a) memory strategy modulation of early (<300 ms) ERPs associated with cue processes and selection of memory strategy to be employed on a trial, and (b) later ERPs associated with volitional memory control (anterior scalp locations) and conscious retrieval (posterior).

#### 1.3.3 Neuroelectric correlates of retrieval suppression (face‐scene pairs)

One challenge for ERP studies of the think/no‐think task is that observed ERPs will initially correspond to the type of cue used (e.g., word, face). Faces as cues have an advantage of increased external validity (e.g., see someone might trigger recall of an event in everyday life), and there are large landmark ERPs associated with the onset of a picture of a face. Rapid visual presentation of a neutral face will typically elicit robust VPP (~170 ms positive peak) and P3 (~400 positive peak) at anterior electrode sites, and robust P1 (~120 ms positive peak) at posterior sites.

Chen et al. (2012; neutral face‐valenced scene picture pairs) found that memory strategy (i.e., think vs no‐think) modulated (a) a posterior P1 (70–140 ms window, think more positive than no‐think) and (b) an anterior N2 (150–260 ms, think more negative than no‐think). Consistent with the earlier literature using word pairs, the posterior P1 was argued to be associated with early visual feature processing of the face cue, and the anterior N2 was argued to be associated with cue‐driven selection of the appropriate memory control strategy. The think/no‐think N2 appears to disrupt the preceding VPP, cutting that ERP short. Chen at al. (2012) also reported an anterior late negativity (LN 380–500 ms window, no‐think trials more negative than think) after the typical face‐related P3, thought to be related to volitional memory strategy. Of greatest importance was their finding of a posterior late positivity component (LC, 500–800 ms window, think trials more positive than no‐think), reflecting greater retrieval on think than no‐think trials. Recently, Zhang et al. (2016, neutral face and valenced scene pairs) replicated the N2 and LC markers of retrieval suppression. The P1 and LN ERPs bear further validation.

### 1.4 Rumination and memory suppression

The *executive deficit hypothesis* (Levy & Anderson, 2008) proposes that cognitive control over memory is an important skill determining the regulation of intrusive memories associated with clinical disorders such as PTSD and depression. This view importantly predicts a relationship between durable memory suppression effects (i.e., negative control effect during think/no‐think task; Anderson & Levy, 2009) and control over intrusive memory (i.e., rumination).

Rumination involves repetitive focusing on causes, situational factors, and consequences of negative life experiences (Nolen‐Hoeksema & Morrow, 1991). An increased tendency toward rumination has been argued to be an important factor in depression and anxiety disorder (Notlen‐Hoeksema, 2000), and evidence from large longitudinal studies of both adolescents and adults supports rumination as a transdiagnostic factor predicting the comorbidity of these mood disorders (McLaughlin & Nolen‐Hoeksema, 2011). The view that rumination involves perseverative focus on certain memories, combined with the proposal that a breakdown in the ability to disengage attention from distracting or negative thoughts or information (Koster, Lissnyder, Derakshan, & De Raedt, 2011), suggests a relationship between memory suppression and rumination.

Studies using the think/no‐think paradigm have consistently found negative correlations between rumination and durable memory suppression as indexed by the negative control effect during the think/no‐think task (Anderson & Levy, 2009). Hertel and Gerstle (2003) found negative relationships between scores on a brief rumination self‐report scale and 2 separate measures of memory suppression on the think/no‐think task. Dieler et al. (2014) reported a negative correlation between brooding (i.e., negative aspects of rumination) and durable memory suppression. Fawcett et al. (2015) tested high and low rumination groups on the think/no‐think task and reported smaller durable memory suppression (negative control effect). Several studies have documented impaired durable memory suppression in groups that also experience reduced ability to control intrusive memories, for example, depression (Hertel & Gerstle, 2003) and PTSD (Catarino, Kupper, Werner‐Seidler, Dalgleish, Anderson, 2015; Hulbert & Anderson, 2018).

### 1.5 Study goals

Building on these reported findings, the current study sought to extend the literature by being the first to document a relationship between ERP correlates of retrieval suppression and individual differences in memory control (i.e., ruminative behaviors). Such a finding would provide important support for the executive deficit hypothesis (Levy & Anderson, 2008), as prior work has reported *negative* correlations between individual differences in memory control (e.g., rumination) and durable memory suppression (e.g., negative control effect, think/no‐thin task; and would add to our knowledge regarding how memory suppression works (Dieler et al., 2014; Fawcett et al., 2015; Hertel & Gerstle, 2003). By extension, we predicted that we would find a negative correlation between rumination and the LC ERP that directly indexes failures of retrieval on no‐think trials (see section 1.3.2 and 1.3.3).

Recently, Zhang, Xie, Liu, and Luo^0^ (2016, neutral face and valenced scene pairs) replicated the N2 and LC ERP markers of retrieval suppression, but not the P1 and LN (see section 1.3.3 for more on ERP correlates of retrieval suppression). The P1 and LN ERPs bear further validation. Moreover, by contrast with Chen et al. (2012) who reported a anterior LN (late negativity) associated with volitional memory control, Bergstrom et al. (2009, neutral word pairs) reported a positivity with the same proposed functionality that should overlap with the face‐related anterior P3 when moving to use of face cues. This suggests that further replication of the LN (Chen et al., 2012) is in order, especially given the proposed role of this ERP as indexing volitional control of retrieval suppression.

## 2 MATERIAL AND METHODS

### 2.1 Design

The current study is a factorial memory experiment with measurement of EEG and cued recall responses measured for face‐picture pairs. The design is a 2 emotional valence (neutral versus negative pictures) × 2 strategy (think versus no‐think) completely repeated measures design. Standard ANOVA models (extra factors are added to the ANOVA model for electrode locations where appropriate) are used to analyze memory effect in the recall and EEG results. This study followed a protocol approved by the University of North Carolina‐Charlotte (UNCC) IRB, and written informed consent was obtained from all participants at time of testing.

### 2.2 Participants

Participants were college students recruited from the UNCC Department of Psychology Research Participation Pool. A total of 33 participants were recruited and received course credit for their participation. Of these, 10 were excluded due to equipment failure, excessive somnolence, noncompliance with task instructions, and/or high movement artifact, leaving a final sample of 23 participants. Two stimulus lists were created by counterbalancing pairing of each target with a randomly chosen male or female cue face across lists. Participants were pseudorandomly assigned to the 2 stimulus list conditions with the constraint of balances sample sizes (*N* = 12, 11, for lists A and B). The mean age of the final sample was 25.3 (*SD* = 9.3). The sample was comprised of 18 females (78.3%) and 5 males (21.7%), and 73.9% were Caucasian, 8.7% were African American, 4.3% were Asian, and 13.1% were of other ethnicities. All participants were native speakers of English and right‐handed, with normal or corrected‐to‐normal vision that allowed them to easily read text and clearly identify and describe pictures presented on the computer screen.

### 2.3 Material and procedures

Participants were administered the Edinburgh Handedness Inventory (EHI; Oldfield, 1971) to control for handedness (only right‐handers included). Participants were also administered the Ruminative Responses Scale (RRS; Nolen‐Hoeksema & Morrow, 1991) which is a self‐report measure of rumination that identifies two rumination subfactors: reflection and brooding, the former represents adaptive rumination and the latter reflects maladaptive rumination (Treynor, Gonzalez, Nolen‐Hoeksema, 2003).

**Figure 1:**
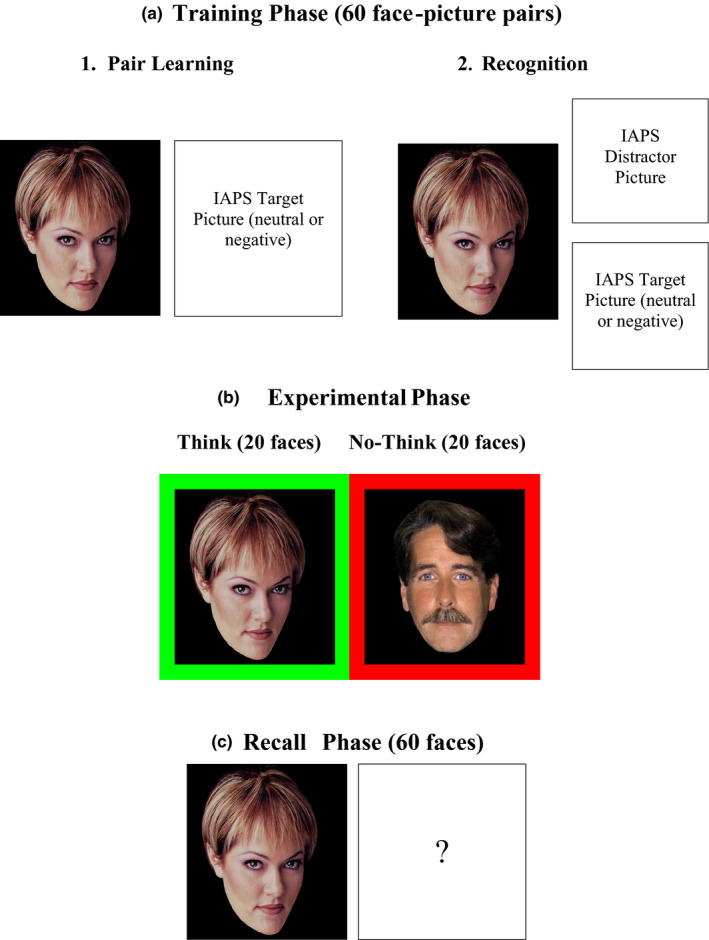
Think–No‐think Paradigm using the International Affective Picture Series (IAPS)

The pictorial stimuli used for the Think/No‐Think task (Figure 1) included 60 photographs of faces, half male and half female, validated to have neutral expressions by Depue et al. (2006). A separate 30 images with neutral emotional valence (e.g., pizza, freeway, baby) and 30 with negative emotional valence (e.g., funeral, scenes of war, electric chair) were also selected from the IAPS (Lang, Bradley, & Cuthbert, 2008). The faces and pictures were pseudorandomly paired to result in four groups of 15 stimuli blocked by sex (male/female) and valence (negative/neutral). Stimuli were presented using E‐prime v1.1 software (Psychology Software tools, Pittsburgh, PA, USA).

During the training phase (Figure 1a), participants learned the 60 face‐picture pairs as they were displayed, one pair at a time, on a computer monitor for 3.5 s followed by a 0.5‐s fixation cross. The cue faces (225 × 225 pixels) were displayed on the left side of the screen, and the neutral or negative target images (225 × 225 pixels) on the right side of the screen (Figure 1a, Panel 1). The pictures were displayed centered vertically on the computer screen and horizontally on either side of the midpoint such that the left edge of the face picture coincided with the left edge of the screen and the right edge of the other picture coincided with the right edge of the screen. Participants viewed subsets of 20 pairs at a time in different random order, and the three subsets were cycled through 3 separate times, with a recognition test at end of each subset of 20 pairs. Each set of 20 pairs had an equal proportion of female/neutral, female/negative, male/neutral, and male/negative face‐picture pairs.

During the recognition test (Figure 1a, Panel 2), participants were shown the cue faces alongside two pictures: one that was originally paired with it and a consistent distractor picked randomly from the stimuli set that remained constant for all 3 recognition cycles. Each picture in the 20 pair subset, therefore, appeared once as a target and once as a distractor in the recognition test. The cue faces were centered vertically on the right side of the screen such that the right edge of the picture coincided with the right edge of the screen. Both the target and distractor pictures were stacked vertically on the left side of the screen such that the left edge of the pictures coincided with the left edge of the screen. The participants were asked to identify the correct face‐picture association by pressing the “T” or “B” button on the response box if the correct target image was on top or bottom of the screen. The intertrial interval presented a gray (neutral) fixation cross for 0.5 s. For each participant, each set of 20 pairs was cycled through three times to overtrain them on the pairs as previous research indicated that the average number of training cycles required to learn the face‐picture associations is close to two (*M* = 1.76, *SD* = 0.61; Depue et al., 2006). Participants with lower than 90% on the third learning cycle were removed prior to data analysis. After the learning phase, participants were fitted with a 40‐channel electrode cap.

During the experimental phase, the participants were comfortably seated in the testing room while their EEG was recorded. The participants viewed 40 of the 60 cue centered vertically and horizontally on the computer screen. Half the faces were from the think condition and half the no‐think condition. For both conditions, a trial consisted of a face framed by a colored border (30 x 30 mm) presented on a computer screen for 3.5 s followed by 0.5 s intertrial interval represented by a gray fixation cross. The border color was varied across trials to signal which strategy a participant should use: green for think trials and red for no‐think trials (Figure 1b). For think trials, participants were instructed to concentrate on the memory of the target picture previously associated with the cue face, or for no‐think trials, to try to prevent recall of the previously associated picture. For each condition (think/no‐think), participants viewed 20 faces 10 times each. After every 2 cycles, a one‐minute break was given to the participants to rest their eyes. Of the original 60 pairs, 20 were assigned to a baseline condition and not shown in the experimental phase.

During the final recall phase (Figure 1c), participants were shown each of the 60 faces for 3.5 s each followed by an intertrial interval of 0.5 s represented by a gray fixation cross. Between each cue face stimulus, they were asked to describe the correct target image in two to three words. Their verbal responses were recorded by the researcher and provided a behavioral measure of cued recall accuracy to assess suppression effects. These descriptions were then scored correct or incorrect by two independent judges (inter‐rater reliability was 0.98). Differences in scores allotted by these two independent judges were adjudicated by a third blind judge. Following the completion of the final test phase, the participant was debriefed.

### 2.4 EEG recording and analysis

Continuous EEG was recorded from 40 Ag/AgCl electrodes embedded in a 40‐channel Neuroscan Quik‐Cap. Electrical oculogram (EOG) was recorded by additional electrodes positioned above and below the left eye (vertical movements), and on the outside edge of the right and left eye (horizontal movements). An additional reference electrode was positioned on the electrically neutral tip of the nose. EEG signals were amplified by a 40‐channel Neuroscan NuAmps amplifier at a sample rate of 500 samples per second. Electrode impedances of 5 kΩ were obtained at all active sites. Data acquisition and postacquisition processing was performed using Scan 4.3 software. Continuous EEG was filtered offline with a bandwidth of 0.1 to 70 Hz, with a gain of 19. Continuous EEG recordings were partitioned into epochs (−200–850 ms) time‐locked with the presentation of each face cue and baseline corrected using the −200 to 0 ms time window as baseline. Epochs were manually inspected and marked as bad in the presence of overwhelming electrical artifact, and epochs with observed potentials outside the ±70 μV range were automatically rejected. The remaining epochs were averaged by group based on the cross of valence (negative versus. neutral) by cognitive strategy (think versus. no‐think).

Four target electrode sites of interest were chosen to be consistent with previous ERP studies of memory suppression (e.g., Bergstrom et al., 2009; Bergstrom et al., 2007; Chen et al., 2012): electrodes F3 and F4 (left and right frontal sites, respectively) and electrodes P3 and P4 (left and right parietal sites, respectively). Averaged ERP waveforms for each of the 4 stimulus conditions, at each electrode of interest, were visually inspected and time windows for assessment of the parietal P1 (100–150 ms), frontal P3 (350–450 ms), and parietal LC (450–550 ms) were identified. Mean area under the curve (equivalent to mean amplitude as areas under the baseline are given negative values) were computed for each participant, for each experimental condition, at each electrode of interest and timewindow of interest. There was no visually discernable N2 (typically peaks at 200 ms poststimulus) effect at the frontal sites of interest, this ERP component was not analyzed.

## 3 RESULTS

### 3.1 Recognition accuracy

Recognition accuracy scores derived from behavioral responses were calculated for the total training phase and for each of the three subdivisions. Overall mean recognition accuracy across all participants and repetitions was 96.2% (*SD* = 3.8%), indicating that participants succeeded in learning stimulus pairings. As expected, mean recognition accuracy was lowest after the first training block (*M* = 91.9%, *SD* = 9.3%), but was similar across the second (*M* = 98.5%, *SD* = 1.9%) and final (*M* = 98.4%, *SD* = 1.8%) training blocks.

### 3.2 Rumination scores

Mean total RRS score for all participants was 37.65 (*SD* = 8.94) on a scale of 22 to 88. On a scale of 5 to 20, the mean score for all participants on the Reflection subscale was 9.48 (*SD* = 3.48) and on the Brooding subscale was 8.65 (*SD* = 2.21).

### 3.3 Behavioral data

**Figure 2:**
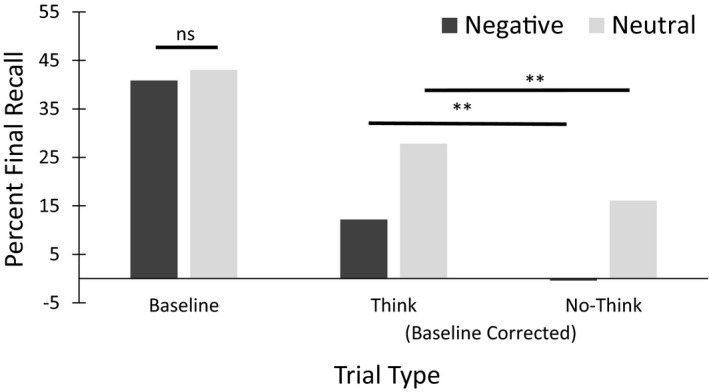
Mean final cued recall percent for negative and neutral baseline face‐picture pairs (on left) and (baseline corrected) face‐picture pairs (on right). Concentrating on the baseline‐corrected final cued recall (on right), (a) neutral pictures yielded significantly greater cued recall than negative pictures (*p *< .01), and this same comparison was significant for each valence in isolation (**p's < .01), (b) no‐think trials did not yield significant cued recall below baseline, and (c) neutral pictures yielded significantly greater cued recall than did negative pictures

Recorded verbal responses on the cued recall test phase were scored correct or incorrect by two independent judges (inter‐rater reliability was 0.98). Differences in scores were adjudicated by a third blind judge. Baseline‐corrected recall scores provided behavioral data for analyses (Figure 2). Total recall scores were corrected for baseline learning by subtracting the baseline recall rate for the 20 baseline stimuli, controlling for valence, from the observed recall rate for the experimental conditions (think and no‐think). Baseline scores reflect the amount of forgetting that could be expected from passive memory decay over the course of the study. The scores were analyzed using a 2 valence (neutral versus. negative) × 2 strategy (think versus. no‐think) ANOVA, with both factors manipulated within subjects. There was a main effect of valence with more items being recalled above the baseline in the neutral condition (*M* = 21.9%, *SE* = 3.1%) than the negative condition (*M* = 6.3%, *SE* = 4.4%), *F* (1, 21) = 9.84, *p* < .01, partial η^2^ = 0.32. A main effect of strategy was also evident as there was a significant difference between the number of items recalled above the baseline in the think (*M* = 20.1%, *SE* = 3.4%) versus no‐think conditions (*M* = 8.1%, *SE* = 2.9%), *F* (1, 21) = 22.66, *p* < .01, partial η^2^ = 0.52. However, there was no significant interaction effect observed (*p *> .19). The think versus no‐think comparison was significant for each valence in isolation (*p*'s < 0.01). Moreover, the baseline‐corrected neutral no‐think mean percent correct was significantly positive (*p* = .03), but the baseline‐corrected mean negative no‐think mean percent correct was not significantly different from 0 (*p *> .50).

Because neither of the no‐think conditions yielded significantly negative baseline‐corrected means, there was no significant negative control (i.e., memory suppression) effect for no‐think trials. However, there was a significant think/no‐think differential (i.e., total control effect), indicating modulation of memory control strategies led to differential final cued recall.

### 3.4 ERP data

**Figure 3:**
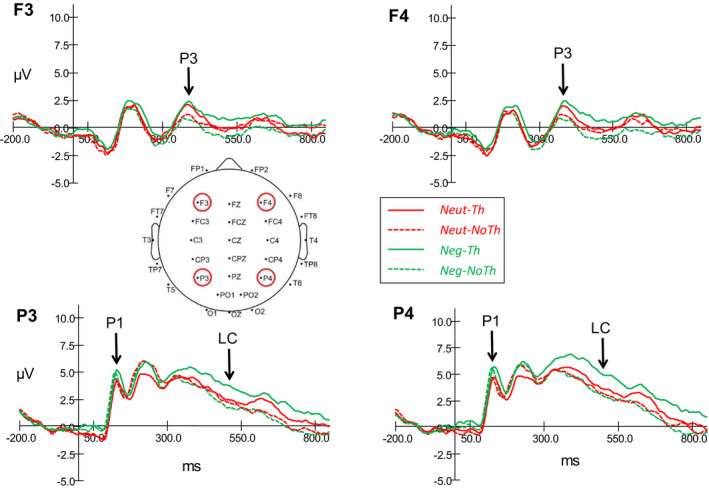
Grand mean ERPs for the four experimental conditions (neutral‐think, neutral‐no‐think, negative‐think, negative‐no‐think) at all four electrode sites (F3‐left frontal, F4‐right frontal, P3‐left parietal, P4‐right parietal)

**Figure 4:**
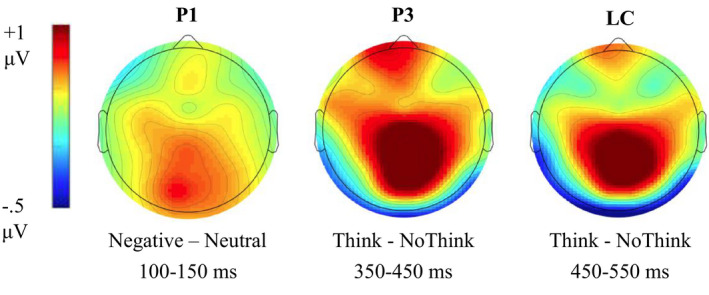
Scalp distribution for comparisons of interest in each time window of interest: P1 (100–150 ms), P3 (350–450 ms), LC (450–550 ms)

For each individual, ERP results to face cue images were quantified by area under curve measures at each electrode site of interest (F3, F4, P3, & P4) for each combination of valence by strategy at time windows of interest (P1, 100–150 ms, P3, 350–450 ms, LC, 450–550 ms). The area measures were submitted to 2 valence (negative, neutral) x 2 strategy (think, no‐think) x 2 laterality of electrode site (left, right) repeated measures ANOVAs. ERP waveforms at the four electrode sites of interest are depicted in Figure 3. Scalp plots of the voltage effects of interest are presented in Figure 4. The left panel presents the negative minus neutral trial voltage difference in the P1 (100–150 ms) time window, and indicates a primarily posterior scalp distribution. The middle panel presents the think‐minus no‐think voltage difference in the P3 (350–450 ms) time window and indicates distinct frontal and posterior foci. The right panel presents the think‐minus no‐think voltage difference in the LC (450–550 ms) time window and indicates a primarily posterior scalp distribution.

#### 3.4.1 Posterior P1 (100–150 ms)

The posterior P1 (positive ERP at parietal electrode sites of interest, P3 & P4, see Figure 3 for map of electrode sites) is thought to reflect attention to sensory characteristics of the stimuli (e.g., face cue). There was a main effect of valence with a greater positive mean ERP area in the negative condition (*M* = 198.2, *SE* = 35.7) than the neutral condition (*M* = 162.0, *SE* = 33.5), *F*(1, 22) = 9.17, *p* = .006, partial η^2^ = 0.29. There were no other significant interaction effects observed (all *F*’s < 3.04 and *p's *> .09).

#### 3.4.2 Anterior P3 (350–450 ms)

The anterior P3 (positive ERP at frontal electrode sites of interest, F3 & F4, see Figure 3 for map of electrode sites) results demonstrated emergence of ERP activity associated with the exercise of conscious control. There was a main effect of strategy with a greater positive mean ERP area in the think condition (*M* = 158.0, *SE* = 57.2) than the no‐think condition (*M* = 61.1, *SE* = 39.9), *F*(1, 22) = 7.32, *p* = .013, partial *η* ^2^ = 0.25. No other main effects or interactions were significant.

#### 3.4.3 Posterior LC (450–550 ms)

**Figure 5:**
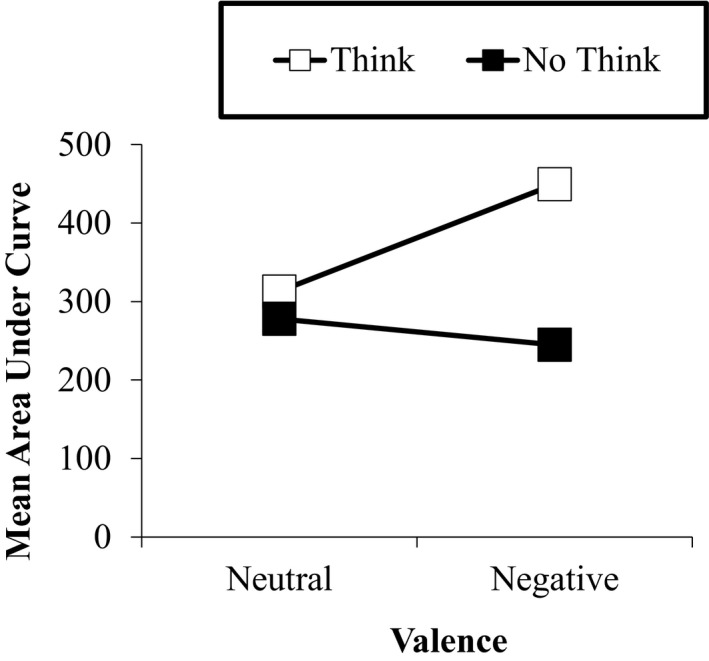
Mean area under ERP for posterior LC (450–550 ms postcue), broken down by valence and cognitive strategy. Significant (*p *< .05) interaction of strategy and valence such that mean area was reduced for no‐think trials in relation to think trials to a greater extent for negative target pictures. Post hoc comparisons at each valence revealed a significantly reduced no‐think mean versus think mean for negative target scenes (*p* < .01) but not for neutral target scenes (*p *> .05)

The posterior LC (Positive ERP at parietal electrode sites of interest, P3 & P4, see Figure 3 for map of electrode sites) is thought to be related to retrieval success and to further processing of retrieved target (Bergstrom et al., 2007, 2009; Chen et al., 2012; Rugg, 1995). There was a main effect of strategy with a greater positive mean ERP area in the think condition (*M* = 381.1, *SE* = 60.7) than the no‐think condition (*M* = 261, *SE* = 56.4), *F*(1, 22) = 4.56, *p* = .044, partial *η* ^2^ = 0.17. There was a main effect of laterality as there was a larger positive mean ERP area on the right electrode site (*M* = 375.7, *SE* = 51.0) compared to the left (*M* = 266.8, *SE* = 58.6), *F*(1, 22) = 8.19, *p* = .009, partial *η* ^2^ = 0.27. There was also an interaction between valence and strategy, *F*(1, 22) = 4.64 *p* = .043, partial η^2^ = 0.17, with reduced mean ERP area for no‐think trials was greater for negative than for neutral target pictures (see Figure 5). Further analysis of the strategy effect at each level of valence revealed a significantly larger area under the ERP for think than for no‐think trials for the negatively valenced pictures, *F*(1, 22) = 9.89, *p* = .005, partial *η* ^2^ = 0.31, but not for the neutral valenced pictures (*p *> .05).

### 3.5 Correlational analyses with rumination scores

In order to explore if rumination scores were significantly related to memory inhibition, the data were analyzed using Pearson correlations. We compared rumination (all three rumination scores: Total RRS, negative Brooding, & positive Reflection) with behavioral recall in the no‐think category, strategy differences in behavioral recall between think and no‐think categories, and area measures for Window 5 (LC) at parietal electrodes (i.e., P3 & P4) for no‐think trials and strategy (think‐minus‐no‐think) differences. We also added post hoc correlations between rumination scores and final cued recall for think and no‐think trials in isolation (see Table 1 for correlations) to allow for better interpretation of the planned correlations. There was a positive correlation between RRS‐Brooding and the combined (across electrodes P3 & P4) posterior LC ERP think/no‐think difference, *r*(23) = 0.48, *p* = .021. This correlation is opposite in direction to our prediction (see Discussion for more). There was also a nonsignificant positive correlation, *r*(23) = 0.30, *p *> .05 between RRS‐Brooding and the final cued recall think/no‐think difference (see Discussion).

**Table 1:** Pearson's correlation between rumination and the posterior LC, and baseline final recall (*N* = 23)

	RRS scores		
	Brooding	Reflection	Total
ERP LC			
Think/No‐think Diff	0.48*	0.24	0.28
No‐think	−0.15	−0.31	−0.10
Think	0.30	−0.07	0.17
Final Recall			
Think/No‐think Diff	0.30	−0.09	0.18

* *p* < .05.

## 4 DISCUSSION

Previous research has indicated that control mechanisms in the brain can be activated to actively suppress completion of a memory retrieval initiated by a memory cue during the think/no‐think task (Anderson & Green, 2001; Bergstrom et al., 2007; Depue et al., 2007). The executive deficit hypothesis (Levy & Anderson, 2008) proposes that retrieval suppression is an important aspect of a more general cognitive control system that has important implications both for individual differences in the regulation of intrusive memories (e.g., following trauma) as well as clinical groups where this is a significant deficit such as depression (Hertel & Gerstle, 2003) or PTSD (Catarino, Kupper, Werner‐Seidler, Dalgleish, & Anderson, 2015). The primary goals of the present study were to (a) to replicate and extend evidence for ERP markers of stages of memory control and memory retrieval processes during the think/no‐think task, and (b) provide evidence for a link between individual differences in rumination and an ERP marker of retrieval suppression. We replicated 3 of 4 memory strategy related ERPs previously reported (Bergstrom et al., 2007, 2009; Chen et al., 2012; Zhang et al., 2016) for variations of the think/no‐think task, the posterior P1 (100–150 ms), the anterior P3 (350–450 ms), and the LC (450–550 ms), but failed to replicate the anterior N2 (150–260). Moreover, our observed P1 and P3 differed somewhat from those reported by Chen et al. (2012) with a similar think/no‐think task. We also provide the first report of a relationship between an ERP correlate of retrieval suppression, the LC, and individual differences in ruminative behaviors, but this correlation was in the opposite direction predicted.

### 4.1 P1

Previous ERP studies of face perception have found an early positivity (P1) over posterior scalp locations peaking 100–150 ms postpresentation (Olivares, Iglesias, Saavedra, Trujillo‐Barreto, Valdes‐Sosa, 2015). We observed a posterior P1 (100–150 ms) that was greater for negative than for neutral trials during the think/no‐think phase (face cues presented with instructions to either allow or not allow the associated scene to come to mind). We did not find significant modulation of the P1 by memory control strategy or by an interaction of memory control strategy and valence. By contrast, the posterior P1 has been found to be more positive for think than for no‐think trials in one previous study (Chen et al., 2012) that used emotionally valenced target scenes and neutral face cues, but this was not replicated by Zhang et al. (2016). However, Bergrstrom et al. (2007; paired words) did find an early memory control strategy related posterior ERP that they proposed was related to early visual processing of the cue (color of cue word indicated memory control strategy), but failed to replicate this effect in a follow‐up study (Bergstrom et al., 2009). Further research is warranted on the factors influencing observation of an early posterior ERP related to cue processing during the think/no‐think task.

Our finding that the posterior P1 during neutral face presentation is modulated by emotional valence of an associated item is, to our knowledge, novel in the literature. However, there is a growing appreciation in the literature on face perception that the P1 is sensitive to emotional expressions of faces (e.g., Earls, Curran, & Mittal, 2016). This suggests a couple of interesting possibilities. First, that as the cue face is processed in the cue‐scene version of the think/no‐think task, that partial retrieval of the emotional valence of the target scene may influence visual processing of the neutral expression face cue. Alternatively, neutral cue faces may come to have an associated emotional valence during learning by virtue of associative links to valences target scenes. Further research may want to explore these possibilities.

### 4.2 N2

Both Chen et al. (2012) and Zhang et al. (2016) reported more negative N2 (~200 ms peak) for no‐think than for think trials at frontal sites. The present study failed to observe a discernable frontal N2 effect (see Figure 3). Previous research on the think/no‐think task has indicated participants may vary in their cognitive control strategies (van Schie, Geraerts, & Anderson, 2013) with active memory suppression and mental substitution (try to think about something else during no‐think trials) being the 2 major ones. The frontal N2 has been argued to be related to early cue‐driven selection of a suppression strategy (Bergstrom et al., 2009), indicating that our participants may have used substitution rather than active suppression more often than the participants in these other studies. Another factor that may have reduced our ability to observe a frontal N2 was our use of a nasal reference electrode location as opposed to the more common combined left/right mastoids (behind the ear). More research on the influence of reference electrode placement on the N2 during cued retrieval is warranted.

### 4.3 P3 versus Late Negativity

Our results yielded a frontal P3 (positivity, 350–450 ms) that was greater for think than for no‐think trials. Bergstrom et al. (2009; paired words) reported evidence that this late (300–500 ms) anterior effect, that began coincident with a P3‐like ERP in the overall average, was related to volitional retrieval suppression control as it was modulated by memory control strategy (i.e., think versus no‐think) and not by learning outcome (i.e., no‐think items learned versus not learned during the initial training phase). While Chen et al. (2012, face‐scene pairs) reported a similar ERP differential centered on a late negativity (380–500 ms), visual inspection of their results reveals clear separation of ERP curves for think versus no‐think trials at the preceding P3‐like potential (~320 ms peak). It is unclear if this was not reported due to statistical nonsignificance, or due to a priori focus on the late negativity deflection. Bergstrom et al. (2007, paired words) found no memory strategy effect for their P3‐like deflection at anterior electrode sites, but memory control strategy ERP effect began clearly coincident with a late negativity. Overall, the literature supports a late 300–500 ms anterior volitional memory control strategy ERP, but the factors that lead this to emerge earlier during a P3 versus later during a late negativity require further research.

Additionally, our use of emotionally valenced picture targets allowed us to look for the influence of emotion processing on this ERP component. However, our P3 was not modulated by target scene valence, or by a memory strategy by valence interaction. The late negativity effect reported by Chen et al. (2012) was modulated by a memory strategy by valence interaction. This suggests that the memory control strategy may appear prior than valence‐related processing. Visual inspection of the ERPs at our frontal (F3, F4) sites suggested a trend toward a memory control by valence interaction in the later 500–600 ms time window, but a post hoc statistical comparison failed to yield a significant effect (*p *> .05). More work needs to be done to determine if there are 2 temporally separable effects or just one.

### 4.4 LC

The present study found an interaction of emotional valence and memory strategy for the posterior LC (450–550 ms), such that think trials had a greater positive area than no‐think trials, and this think/no‐think difference was greater for negative than neutral trials (see Figure 5 for pattern of interaction). In fact, only the negative trials yielded a significant think‐minus‐no‐think difference. The pattern of the interaction suggests that negative target scenes received greater retrieval suppression. Zhang et al. (2016) found a similar interaction using a similar set of neutral face cues and emotionally valenced target pictures. However, the think‐minus‐no‐think difference was significant for both negative and neutral trials. Of interest to our results is the group comparison reported in this study. Zhang and colleagues also reported a significant group difference in this interaction such that the strategy effect was much greater for negative trials than for neutral trials in a group of depressed individuals. Whereas the nondepressed group yield similar strategy effects for both negative and neutral trials.

### 4.5 Rumination & Retrieval Suppression

Another goal of this study was to observe the role of ruminative styles of thinking on memory suppression as measured by the RRS (Nolen‐Hoeksema & Morrow, 1991). This provides a clinically useful corollary between this study and management of negative memories. We predicted that high ruminators would not be able to exert memory control processes as efficiently as low ruminators resulting in smaller differences in scores across the four experimental conditions for the former. Results, however, indicated the opposite relationship between RRS scores and ERP suppression effects. This relationship was contrary to what we expected and suggests that high brooders show better suppression of presented material. This relationship also contradicts Hertel and Gerstle’s (2003) findings demonstrating a negative correlation between suppression effect and depressive symptoms, specifically depressive rumination.

One explanation for this pattern is that, because our sample consisted of college students who were not necessarily clinically depressed, the high scorers on the brooding subscale probably do not capture maladaptive patterns of brooding in our study. Instead, it is likely that we are observing a pattern of individuals engaging in moderate levels of rumination being most adept at actively suppressing memories. Similar findings of a relationship between higher reported trauma were found with better suppression scores in nonPTSD patients (Hulbert & Anderson, 2018), while individuals diagnosed with clinical levels of PTSD demonstrate poorer memory suppression (Catarino et al., 2015). This might suggest that moderate (and not severe) levels of aversive experiences may make individuals better at suppressing negative stimuli due to prior experiences of doing so or a process of resilience. Further research would, therefore, be warranted to examine if specifically training patient groups on memory control strategies in conjunction with distress reduction of trauma or mood symptoms would improve their suppression of unwanted past memories.

### 4.6 Durable memory suppression

The present study failed to find durable memory suppression effects as assessed by the negative control effect, below baseline final cue recall of no‐think items. Negative control effects are not always observed in variations of the think/no‐think task (e.g., Bergstrom et al., 2007; Bulevich et al., 2006; Hertel & Gerstle, 2003), Dieler et al. (2014) list another 7 studies with similar failures to find negative control effects. In fact, there is an ongoing research effort seeking to document variables that affect the likelihood of observing negative control effects (e.g., Dieler et al., 2014). We did observe robust total control effects, greater final cue recall for think than no‐think trials (~15%, see Figure 2) that was constant across target scene valence.

While the negative control effect (Anderson & Levy, 2009) was the original gold standard for durable memory suppression, failures to observe this effect consistently (e.g., Dieler et al., 2014) have led to the development of an independent cue modified version of the think/no‐think task (Levy & Anderson, 2008) that, in conjunction with negative control effects, can provide better estimates of durable memory suppression effects in studies that have such effects as their focus of study. The present study was designed to examine neurocorrelates of retrieval suppression, and the indirect cue procedure is difficult to use with pictures of scenes as target items in the think/no‐think task.

Our baseline‐corrected final recall did not exhibit an interaction between memory control strategy and valence, there were equivalent total control effects for negative and neutral target scenes. The original study to use the neutral face cues and emotionally valences target scenes, Depue et al. (2006) reported a memory control by valence interaction such that the total control effect was greater for negative than for neutral trials. By contrast, Chen et al. (2012) reported the opposite interaction, with total control effects smaller for negative than for neutral. We are not sure what to make of this inconsistency, again, the problem may be that baseline final recall is sensitive to variations in experimental procedure, group differences, and individual differences as suggested by Dieler et al. (2012).

## 5 CONCLUSION

In summary, the present study provides further evidence for think/no‐think related ERP effects previously reported in the literature: (a) a frontal P3 (350–450 ms) alerting signal in preparation of cognitive control of memory retrieval modulated by strategy but not emotional valence, (b) a later posterior LC (450–550 ms) modulated by a combination of strategy and emotional valence. The present study extends evidence regarding an early posterior P1 by finding that it is modulated by emotional valence, not memory strategy as in earlier reports (e.g., Chen et al., 2012). Our results also provide the first report of a relationship between rumination and the posterior LC thought to index retrieval success (e.g. Curran et al., 2006). Considering that this study was able to establish these findings using a college population, the next step would be to research clinical populations using this paradigm. One direction would be to examine the role of depressive rumination in greater detail by including more diagnostic scales of depression in the design as well as testing a larger sample. Our current findings that suppression effects at parietal electrode sites are positively related to rumination indicate that suppression may possibly be a learned response. Another interesting future direction is to examine if retrieval suppression mechanisms are somehow disrupted for clinical disorders like PTSD and OCD that are characterized by intrusive thoughts, especially when the stimuli used are negatively valenced. As mentioned before, such research could have promising clinical implications as identifying such a deficit may propel research in the direction of finding ways to teach individuals to enhance cognitive control mechanisms before their symptoms become debilitating.

## CONFLICT OF INTEREST

The authors do not have any potential conflict of interests to disclose.

## AUTHOR CONTRIBUTIONS

Conceptualization **–** AN, MF; Methodology – AN, JE, MF; Investigation – AN, JE; Formal Analysis – AN, JE; Resources – MF; Writing – AN, MF; Visualization – AN, JE; Supervision – MF.

### Peer Review

The peer review history for this article is available at https://publons.com/publon/10.1002/brb3.1748.

## Data Availability

The data that support the findings of this study are available from the corresponding author upon reasonable request.
